# Dietary Phosphorus and Calcium Utilization in Growing Pigs: Requirements and Improvements

**DOI:** 10.3389/fvets.2021.734365

**Published:** 2021-11-24

**Authors:** Marion Lautrou, Agnès Narcy, Jean-Yves Dourmad, Candido Pomar, Philippe Schmidely, Marie-Pierre Létourneau Montminy

**Affiliations:** ^1^Département des sciences animales, Université Laval, Quebec, QC, Canada; ^2^UMR Modélisation Systémique Appliquée aux Ruminants, INRA, AgroParisTech, Université Paris-Saclay, Paris, France; ^3^UMR Biologie des oiseaux et aviculture, INRA, Nouzilly, France; ^4^PEGASE, Agrocampus Ouest, INRA, Saint-Gilles, France; ^5^Agriculture et Agroalimentaire Canada, Sherbrooke, QC, Canada

**Keywords:** phosphorus, calcium, mitigation, requirements, environment, swine

## Abstract

The sustainability of animal production relies on the judicious use of phosphorus (P). Phosphate, the mined source of agricultural phosphorus supplements, is a non-renewable resource, but phosphorus is essential for animal growth, health, and well-being. P must be provided by efficient and sustainable means that minimize the phosphorus footprint of livestock production by developing precise assessment of the bioavailability of dietary P using robust models. About 60% of the phosphorus in an animal's body occurs in bone at a fixed ratio with calcium (Ca) and the rest is found in muscle. The P and Ca requirements must be estimated together; they cannot be dissociated. While precise assessment of P and Ca requirements is important for animal well-being, it can also help to mitigate the environmental effects of pig farming. These strategies refer to multicriteria approaches of modeling, efficient use of the new generations of phytase, depletion and repletion strategies to prime the animal to be more efficient, and finally combining these strategies into a precision feeding model that provides daily tailored diets for individuals. The industry will need to use strategies such as these to ensure a sustainable plant–animal–soil system and an efficient P cycle.

## 1. Introduction

Phosphorus (P) is an essential element for all living beings, as it is a key component of nucleic acids and energy transfer molecules (adenosine triphosphate, creatine phosphate) and a major mineral component of bone (1). The element P is found in animals as orthophosphates. This is the circulating form of P. Adequate amounts must be provided in livestock diets to ensure animal growth and health. To date, producers have used inorganic phosphate, a limited and non-renewable resource that will be depleted within 100–200 years at current rates of extraction (2). As a commodity mineral, its price is volatile (3). Of greater concern is that P is not absorbed completely from any diet, and in the case of monogastric livestock farming, phosphorus-laden run-off can pollute and cause eutrophication of waterways, which can lead to growth of toxic nitrogen-fixing algae or cyanobacteria (4). This compromises the sustainability of pig farming, which has become highly concentrated in certain regions of several pork-producing countries. In swine production, to avoid an excess of P, the cost of transporting P-rich manure for use as crop field fertilizer can be high and the cost of treating it can be prohibitive; rational and efficient use of P is therefore essential.

Calcium (Ca) is the most abundant mineral in the body (1, 5) and is indispensable for bone mineralization, muscle contraction, and nerve impulse propagation. It is not an expensive element in livestock feed, it is abundant, and it does not represent a threat to the environment. However, as absorption and utilization of P in growing pigs is related to that of Ca, P and Ca requirements must be studied together. Insoluble and indigestible Ca–P complexes can form in the intestines (6, 7). Ca and P deposits in bone are co-dependent. If discharges of phosphorus are to be minimal and its efficiency of utilization must be maximized, its supply must be matched as closely as possible to the requirements of the animals. To achieve this, the actual usable P content of feedstuffs and the animal physiological requirement both must be estimated accurately and precisely. Both global and factorial methods have been used to estimate the Ca and P requirement.

P and Ca requirements can be estimated to maximize growth performance, keep P rejection minimal and/or maximize bone mineralization. Novel approaches in development aim to improve the digestive and/or metabolic utilization of P, thereby decreasing P excretion. The best-known example is the use of phytases, which facilitate the digestion of plant P as phytic acid, the phosphoric ester of inositol, a compound found in many plants and poorly absorbable by pigs. The new generation of phytases makes this strategy even more attractive. The depletion–repletion, a strategy less well known, consists of reducing P and Ca input below the animal's requirements over some period of growth and then increasing the supply as needed (8). This strategy can increase the animal's P digestive efficiency and metabolic utilization in growing pigs; thus it overall decreases in P intake and excretion while maintaining growth and bone mineralization (9, 10). Finally, a mechanistic model approach predicts bone ash and then P and Ca requirement (11, 12) and does not estimate the P and Ca requirement for bone directly from protein. This is an interesting multicriteria approach to mitigate P impact that will be essential for P precision feeding (13). The objective of this paper is to review the latest P and Ca assessment of bioavailability methods for evaluating the nutritional values of feed ingredients for pigs and estimating precisely P requirements, as well as, describing innovations and promising strategies to decrease the P excretions by growing pigs.

## 2. Precisely Assess Bioavailability and Evaluating the Nutritional Values of Feed Ingredients

### 2.1. Dietary Forms of Phosphorus and Calcium

#### 2.1.1. Plant Phosphorus and Calcium

**Figure 1:**
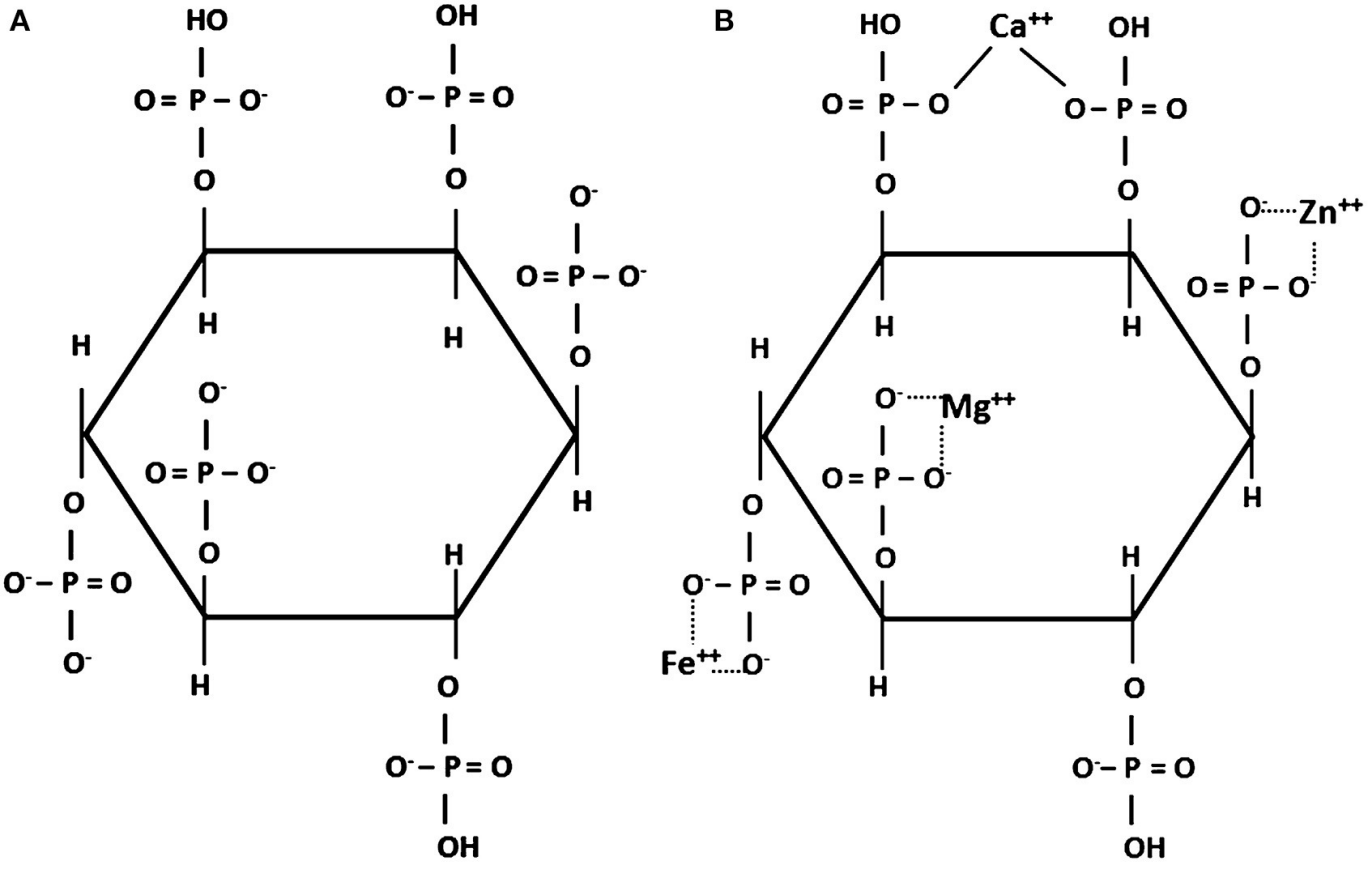
**(A)** Structure of phytic acid at neutral pH (14); **(B)** phytate chelate with different cations. (14).

Phytic acid is synthesized in plants by phosphorylating inositol in any or all possible positions. It can thus bear 6 phosphate groups (IP_6_) as shown in Figure 1, or a smaller number (IP_5_-IP_1_). The main form found in feed ingredients of plant origin is IP_6_ (15, 16). Phytic acid plays a key role in plant metabolism by constituting a reserve of P and chelating other minerals, whereas inositol is used in cell wall formation (17). Phytic acid is present in all plant-based ingredients (18), in which it accounts for 50–80% of the P content (19, 20) and is found almost entirely in the form of salts called phytates, primarily with Ca, Fe, Zn, Mg, K, and Mn. Phytates are solubilized at gastric pH, whereas the higher pH of the small intestine is conducive to their re-formation or *de novo* complexation thus decreasing the absorption of minerals and trace elements (21, 22). *In vitro*, phytic acid forms its most stable salts with Cu, Zn, Ni, Co, Mn, Fe, and then Ca (23, 24). Ca rarely makes up more than 1% of plant dry matter (20) and its absorption is decreased by phytate formation, but this can be countered somewhat by using phytases (see section 4.2), which break down phytates that are in solution (25–27). The cation-binding ability of phytic acid declines as phosphate groups are removed (21). Phytates form insoluble complexes also with proteins, amino acids, and starch and thereby decrease the digestibility in the small intestine and utilization of these nutrients (18).

#### 2.1.2. Mineral Phosphorus and Calcium

P is usually added to pig diets as dicalcium phosphate, which represents 60% of the feed phosphates used in the European Union; monocalcium and monodicalcium phosphates are also used (28). Magnesium, calcium-magnesium, ammonium, and sodium phosphates are also available for use in livestock feed (28–30). To minimize excreted phosphate, which becomes pollution, the most digestible phosphates are preferred, although price also is considered. The first and foremost criterion is to meet narrow technical specifications in terms of composition and physicochemical stability. Phosphates can be classified according to their solubility in 2% citric acid solution. This test does not indicate real digestibility but makes it possible to rank different products (29). A feed-grade phosphate must be at least 95% soluble in 2% citric acid and in alkaline ammonium citrate (28, 31). For monocalcium phosphates, the solubility in water must be greater than 80%, and for monodicalcium phosphate, greater than 50% (28). Monocalcium phosphate is more digestible than monodicalcium phosphate, which is more digestible than dicalcium phosphate (28, 29). Dicalcium phosphate dihydrate is more digestible than the anhydrous form. The final criterion for judging the quality of a feed-grade phosphate is its level of undesirable substances such as arsenic, cadmium, lead, fluorine, or mercury, and dioxins (28, 29).

The inorganic Ca supplements most used in pig farming are calcium phosphates (32) and carbonates supplied in the form of limestone, a mineral that contains calcium carbonate and dolomite and which varies in Ca content (35–38% Ca; (19)). The bioavailability of the Ca in these sources is in the 90–100% range of calcium carbonate (CaCO_3_) used as reference (19, 20, 30). Unlike in poultry, carbonate particle size appears to have no significant effect on apparent or standardized Ca digestibility in growing pigs, based on tests with animals in the 10–20 kg live weight range (33, 34). Calcified red algae has been studied due to its solubility at gastric pH. It is 32% more soluble than calcareous Ca at pH 6.7 and 34% more soluble at pH 3.0 (35). Limestone is 100% calcite, whereas CeltiCal (Celtic Sea Minerals) is 65% calcite, 23% aragonite, and 12% valterite (polymorphs of calcite). The greater solubility does not make the Celtic Sea product more digestible than calcium carbonate. Its digestibility in pigs is at best equivalent to that of calcium carbonate [apparent total tract digestibility [ATTD] Ca of 46.7% and 51.2% for calcium carbonate and CeltiCal, respectively (36), 64% for both sources (37), and may be lower: 46.9% of ATTD Ca and 30.5% for calcium carbonate and CeltiCal (38)]. Marine Ca is absorbed poorly in the upper parts of the gut (38); the higher concentration of dissolved cations moreover makes it precipitate more with phosphate or phytic acids, decreasing P digestibility, bone mineralization, and animal growth (35, 36). A highly soluble Ca to P ratio makes precipitation more likely. Nevertheless, adding marine Ca in smaller amounts and using phytase allows proper balancing of the soluble Ca:P ratio and growth performance equivalent to the control group, and quantitatively superior bone mineralization, at least in broiler chicken studies (35). These results show, above all, that Ca and P interact strongly in the digestive system, and how much further study, especially of the soluble Ca:P ratio, is needed to optimize their utilization.

#### 2.1.3. Phosphorus and Calcium of Animal Origin

In addition to inorganic phosphates, meat and bone meal from the rendering industry is also used as a source of P and Ca. Except in Europe, where it is prohibited in livestock feed other than for fish, these by-products are commonly included in poultry diets. Meat and bone meal can be made up of bones and soft tissues but not blood, hair, hoof, horn, skin/leather, stomach and ruminal contents, or excrement. Most meat and bone meal in North America is a mixture of cattle, pig, and poultry by-products (39). It must contain at least 4.0% of P, and the Ca:P ratio must not exceed 2.2 [AAFCO 2011, cited in Sulabo and Stein (40)]. Meat and bone meal is a source of highly available Ca and P (41, 42) but has unpredictable quality and Ca:P ratios, due to differences in raw materials and processes (39). Depending on the source, the P and Ca contents may vary 2–4 times as much as the protein content, the coefficients of variance being, respectively, 20, 22, and 6.2% (40). A negative correlation exists between protein concentration and P and Ca concentration, due to variations in the proportions of soft tissue and bone (40). The most important sources of variation in the composition of meat and bone meal are therefore the origin of the by-products used and the ratio of soft tissue to bone.

When meat and bone meal is fed to pigs, it provides much of the Ca and P in the diet. It is therefore necessary to have a supplier that uses controlled processes and can guarantee Ca and P content. The standardized digestibility of P in meat and bone meal ranges from 55 to 84% and averages 70% (20, 40), falling between the values for inorganic P and materials of plant origin. Standardized Ca digestibility in meat and bone meal is estimated at 77% but can be 82% for poultry meal (43). The apparent Ca digestibility ranges from 55 to 84% (40). The digestibility of P and Ca in meat and bone meal does vary somewhat, due mostly to the P concentration: the higher the P concentration, the lower the Ca and P digestibility. Since the P concentration depends mainly on the proportion of bone in the meal, it may be presumed that the higher the bone-to-meat ratio, the lower the Ca and P digestibility. The apparent digestibility of P in bone meal is in fact about 68 vs. 80% in meat-and-bone meal and 85% in meat meal (44). Hydroxyapatite, therefore, seems to be a less digestible form of P and Ca. This has been validated for P by comparing diets containing different forms of bone meal. The pre-cecal digestibility of P in chickens is lower when it is still in the form of hydroxyapatite than when it has been previously dissolved (45). Although its composition may vary widely, meat and bone meal offers the possibility of recycling, providing sufficient P to livestock without inorganic P from non-renewable sources. At least one study suggests that heat and pressure treatment of bone meal and removal of gelatin may improve P digestibility (45).

### 2.2. Precisely Estimates of Dietary Phosphorus and Calcium Values of Feedstuffs

#### 2.2.1. Total Analyzable Value

Total dietary Ca and P content in feed ingredients are routinely measured by chemical analysis. However, these numbers do not indicate what portion the animals digest or retain or how much will be excreted. Although this method has its drawbacks, it is still the preferred method for Ca, mainly because of the lack of knowledge on Ca bioavailability. Recent work underway is expected to provide a more accurate Ca bioavailability assessment method with standardized digestibility measurements (46). The P system is more precise with different expression modes, which will be described in the following sections.

#### 2.2.2. Relative Bioavailability

Bioavailability, also called availability, was added in the ninth edition of NRC (47). Availability is an indicator of the use of a nutrient based on a predefined criterion, for example, in the case of P and Ca, bone mineralization measured in terms of mineral (ash) content or a biomechanical property such as breaking strength (48, 49). The value is obtained by comparison with a reference that is considered 100% bioavailable, usually monocalcium phosphate. The relative bioavailability of a nutrient in an ingredient is generally expressed as the slope ratio, which is obtained from linear regressions of the criterion vs. nutrient ingested (48). The main disadvantage of this method is that it is not standardized and thus the bone and parameter measured (e.g., ash content and break strength) may differ between studies, so studies are not comparable (46).

#### 2.2.3. Digestibility

The digestibility concept was first used to assess P content of feedstuffs as ATTD in the Netherlands (50) and then in France (19). In 2012, the National Research Council (NRC) proposed another method, like the one used for amino acid and that should be more precise, the standardized total tract digestibility (STTD). Digestibility refers to the quantity of nutrient that is not found in the feces and therefore must have been digested, or, at least, has disappeared from the digestive tract, a definition that must be nuanced according to whether endogenous losses are considered. Unlike other nutrients, the digestibility of P (and of Ca to a lesser extent) is estimated over the entire digestive tract as fecal digestibility. Two reasonable assumptions justify this: (1) P and Ca are absorbed in the cecum and the colon, respectively (51). These play a homeostatic role in maintaining serum P and Ca under conditions of low intake, and (2) for P and Ca in most dietary supplies, there are no difference between fecal and ileal digestibility for true and apparent P digestibility (52, 53), or apparent and standardized Ca digestibility (38) and therefore no interest in estimating ileal digestibility, which is much more difficult and expensive than measuring fecal digestibility (20).

Apparent total tract digestibility (ATTD) of a nutrient in a feed is the difference between the total intake of the nutrient in question and the amount found in the feces (54, 55):


(1) $$\begin{array}{l} \begin{array}{ccc} ATTD_{Ca\;or\;P}(\%) & = & \left[(Ca\;or\;P_{intake}-Ca\;or\;P_{feces})/Ca\;or\;P_{intake}\right] \end{array}\\ \;\begin{array}{cc} \times & 100 \end{array} \end{array}$$

The methods most used to determine apparent digestibility are total feces collection or partial collection in conjunction with an indigestible marker. Apparent P digestibility is still used widely but has the disadvantage of not being additive in feeds composed of several ingredients (55, 56).

STTD considers basal endogenous losses, which represent the minimal loss of a nutrient, independent of feed composition but influenced by dry matter intake (49, 54). These losses were first estimated by regression with extrapolation to zero ingestion of the studied mineral (57). They are now measured by analyzing feces of animals fed a diet free of P or Ca (34, 37, 58–60). Critics of this method point out that Ca metabolism is well known to be regulated through absorption and thus reabsorption of endogenous losses, leading to underestimation of basal endogenous losses (61). Likewise, a P imbalance due to a P-free but Ca-containing feed would probably affect endogenous P losses (62). Further trials are needed to determine whether endogenous P losses should be measured with a P-free and Ca-free diet, or if it is better to measure P losses with some Ca to minimize interference by regulation. Basal endogenous losses of P and Ca fall, respectively, into the ranges of 139–252 mg and 123–670 mg/kg of dry matter intake (DMI) (37, 38, 63, 64). Basal endogenous P losses in pigs have been estimated at 190 mg/kg of DMI by (20) and 6 mg/kg of live bodyweight (BW) by Bikker and Blok (65). Standardized digestibility can then be calculated using the following equation (55):


(2) $$\begin{array}{l} STTD_{Ca\;or\;P}(\%)=[(Ca\;or\;P_{intake}-(Ca\;or\;P_{feces}\\ \;-Basal\;endogenous\;losses))/Ca\;or\;P_{intake}]\\ \;\times 100 \end{array}$$

Standardized digestibility values are considered additive in feeds composed of several raw materials (20, 46). According to this equation and the use of a constant basal P loss of 190 mg/kg of DMI, it is simple to convert values of ATTD digestibility values into STTD values.

True digestibility accounts for total endogenous losses, which include basal and specific endogenous losses. The latter represents the losses above basal endogenous ones, due to specific characteristics of the feed, such as the level of anti-nutritional factors and fiber content (54). No method of direct measurement of true digestibility exists, except the use of radioisotopes that are now banned in many countries. It is therefore determined by regression, using apparent digestibility and ingested quantity of the nutrient (46, 49):


(3) $$\begin{array}{r} Ca\;or\;P_{absorbed}=\left(TTTD\times Ca\;or\;P_{intake}\right)-Total\;endogenous\;losses \end{array}$$

The negative intercept corresponds to total endogenous loss, while the slope of the regression represents true digestibility. Critics of this method point out that for P and Ca, estimates are highly variable, dependent on individuals, often intercept is not different from 0 (53, 66, 67) and influenced by the amount ingested, in violation of the basic assumption of the regression method (66, 67).

Although several studies about Ca digestibility have been completed (34, 37, 40, 52, 59, 66, 68), the Ca requirement continues to be generally expressed as a total requirement (20, 46) due to the lack of data on digestibility in specific feed ingredients. To overcome the non-additivity of apparent digestibility in a mixed feed, most recent studies have focused on standardized digestibility (59). However, basal endogenous Ca losses measured so far are highly variable and appear to depend on feed composition (59). In addition, components such as fiber may have a direct and proportionate positive effect on standardized Ca digestibility (59), as shown in rat studies (69). These last considerations show the interest in evaluating the Ca digestibility of raw materials under the specific conditions in which they will be used, as recommended in chicken for P (70).

#### 2.2.4. Mechanistic Modeling and Meta-Analysis Approach

All the methods described earlier give a unique P and Ca value for each feedstuff regardless of the interactions with other components of the diet. With the objective of precisely estimate the digestibility of dietary P in a complete diet, two approaches have been used by Létourneau-Montminy et al. (71, 72) based on available literature. First, a mechanistic research mathematical model that simulates the fate of dietary P forms, phytate P (PP) and non-phytate P (NPP) from plant, mineral and animal origin, in the gastro-intestinal-tract was developed and evaluated by Létourneau-Montminy et al. (71). The proposed model integrates and predicts the impact of the most relevant physiological processes involved in P digestion and absorption, including P dietary forms, the presence of exogenous phytase, and the dietary concentration of Ca. It also predicts the impact of transit time and pH of the different dietary sections. The output is the standardized P and Ca absorbed. It can be used as a prospective tool to study P digestibility for different feedstuffs and feeding strategies, as well as the effect of specific digestive processes on P digestive utilization. Second, given the large number of publications on P digestibility in pigs, meta-analysis, a statistical method relevant for summarizing and quantifying knowledge acquired through previously published research (73, 74), was chosen to predict P digestibility considering dietary P forms, Ca, and exogenous phytases. Dietary forms are PP and NPP from plant, mineral, and animal (72). This study provided a generic response of ATTD P (g/kg) to variation of PP, NPP, and phytase. Results showed a linear relationship between NPP and digestible P. Both NPP from mineral and animal feedstuffs and NPP from plant are highly digestible (78 and 73%, respectively). A digestibility coefficient of 21% was also found for PP showed that part of the PP is available for absorption without any exogenous phytase supply (75–77). Then microbial phytase improved digestible P given hydrolysis was simulated with a classic enzyme equation, the Michaelis–Menten. Its response depends on PP quantity, its substrate. The addition of 500 FTU of microbial phytase per kg of feed to a diet with 2 g of PP/kg, increased the amount of digestible P by 0.60 g/kg. With 3 g of PP/kg, the amount of digestible P increased by 0.67 g/kg. It is worthy to note that the amount of PP varies little in swine ingredients. Finally, dietary Ca linearly decreases digestible P independently of phytase supply as previously shown when testing different concentrations of dietary Ca crossed with different levels of phytase (78, 79). This simple method allows a prediction of true P digestibility based on chemical analysis of the diet in total P, PP, Ca, and microbial phytase, while NPP is the difference between total P and PP as used in broilers (80).

## 3. Precisely Assess Phosphorus and Calcium Requirements

According to the FAO and WHO (81), a nutrient requirement is defined as the intake level that will meet specified criteria of adequacy without risking deficit or excess. These criteria include an array of biological effects associated with the nutrient. In livestock production, a requirement is defined as the quantity necessary to maximize a production factor such as body growth or bone mineralization. In practice, growth alone is often a poor indicator of mineral status. Tissue analyses should always accompany growth and feed intake data when evaluating mineral adequacy (82). Bone mineralization has long been the standard, but environmental issues have led several countries to review this, giving rise to the notion of growth performance (20). Ca and P requirements may be defined as facilitating growth according to genetic potential while ensuring optimal bone mineralization and keeping environmental risks minimal. In other words, a multicriteria approach to setting nutrient requirements is needed. To respond to these different objectives, global and factorial approaches, and increasingly mechanistic models simultaneously consider the most important variables, including genetics, live weight, and sex.

### 3.1. Global Approach

This method consists of measuring different performance criteria (growth rate, feed conversion ratio, etc.) in herds that have been fed with increasing levels of the tested nutrient. If all the criteria are not satisfied simultaneously, the proper intake is then considered to be the one that optimizes the most important criterion (83). At this time, the digestibility of nutrients was not considered. This approach presents two main disadvantages. The first one is that it is difficult to compare the estimation of nutrient requirements by this approach with the availability or digestibility of the raw material. The second is that, like nutrient availability, the approach does not consider the portions of P and Ca effectively used and does not allow differentiation between the portions released in feces and urine. Global approaches were replaced by factorial approaches in the 1990s.

### 3.2. Factorial Approaches

A more advanced method is the factorial approach, which consists of quantification and the addition of the requirements for each physiological function (e.g., for maintenance and growth). With the emergence of this method came the consideration of the intestinal absorption of minerals. Several factorial methods estimating P and Ca requirements had been proposed such as Jondreville and Dourmad (84), NRC (20), and Bikker and Blok (65). The first of these methods is based partly on studies conducted years ago in France (83, 85) and the Netherlands (86) and is applied widely in France and Europe. The second one is popular in North America. The third one is in fact an update of the Jongbloed et al. (86) method based on data published since then. The P requirements estimated by these methods are presented in Table 1.

**Table 1:** Estimates of P and Ca requirements for growing pigs according to different models.

**Bodyweight**	30 kg				50 kg				70 kg				100 kg			
**ADG**	0.96 kg				1.11 kg				1.17 kg				1.12 kg			
**Feed intake**	1.36 kg				2.14 kg				2.71 kg				3.22 kg			
**Body protein**	4.68 kg				7.08 kg				11.09 kg				15.03 kg			
	**CVB^*a*^**	**NRC^*b*^**	**INRAe^*c*^**	**Lautrou^*d*^**	**CVB^*a*^**	**NRC^*b*^**	**INRAe^*c*^**	**Lautrou^*d*^**	**CVB^*a*^**	**NRC^*b*^**	**INRAe^*c*^**	**Lautrou^*d*^**	**CVB^*a*^**	**NRC^*b*^**	**INRAe^*c*^**	**Lautrou^*d*^**
STTD P (g/kg)	3.77	4.2	-	4.07	2.83	3.07	-	2.85	2.39	2.45	-	2.38	2.01	1.83	-	2.12
ATTD P (g/kg)	-	-	4.0	3.9	-	-	2.98	2.68	-	-	2.5	2.21	-	-	2.1	1.95
Total Ca (g/kg)	9.96	9.03	11.61	8.16	7.53	6.6	8.65	5.9	6.38	5.27	7.26	5.2	5.4	3.93	6.09	5.06
Total Ca:STTD P	2.64	2.15	-	2.00	2.66	2.15	-	2.07	2.67	2.15	-	2.18	2.69	2.15	-	2.39
Total Ca:ATTD P	-	-	2.90	2.09	-	-	2.90	2.20	-	-	2.90	2.35	-	-	2.90	2.59

a *Estimated according to Bikker and Blok (65)*.

b *Estimated according to NRC (20)*.

c *Estimated according to Jondreville and Dourmad (84)*.

d *estimated according to Lautrou et al. (12)*.

In Jondreville and Dourmad (84)'s model, estimation of P and Ca requirements aims for a bone mineralization of 100%. The maintenance requirement corresponds to obligatory urinary losses, because the P requirements are expressed on an ATTD basis, and endogenous fecal losses are already considered. The maintenance P requirements are estimated at 10 mg/kg of BW (85). The requirement for growth is assessed based on the average daily gain. Finally, the total Ca requirement is estimated according to a fix ratio of 2.9 with the ATTD P requirement.

The NRC (20) considers that P and N retention are highly correlated, and that this correlation is affected little by animal genetics or sex. According to their model, maximal P retention in growing pigs is dependent on body protein. Endogenous basal losses in the gastrointestinal tract are estimated at 190 mg/kg of DMI, to express the P requirements in STTD, and minimal urinary loss at 7 mg/kg of BW. Finally, growth performance is maximized by considering the standardized digestible P requirement to be 85% of the level that maximizes body P retention. The total Ca requirement is set at 2.15 times the standardized P requirement.

In Bikker and Blok (65), Ca and P requirements are estimated independently and aim for a bone mineralization of 100%. The requirement is the sum of the Ca or P retention and the maintenance requirement. An allometric relationship links body Ca and P retention to animal empty body weight gain. The maintenance requirement includes the obligatory urinary loss and the minimal endogenous loss. Basal fecal endogenous losses of P and Ca are set at 6 mg and 8 mg/kg of BW, respectively, and obligatory urinary losses are estimated to be 1 mg and 2 mg/kg of BW. These unavoidable losses are low under conditions of low P or Ca supply, and become greater as the supply increases. The utilization efficiency of the absorbed P and Ca is therefore set at 98%. The Ca and P requirements are first estimated as standardized before applying a digestibility coefficient of 58% to the Ca requirement for expression as total Ca. In this model, as in Sauveur and Perez (83) Ca requirement is estimated according to a factorial approach based on digestible Ca and expressed on a total basis assuming 45–50% Ca digestibility. This permits adaptation of the Ca:digestible P requirement according to animal weight and performance. The same approach was recently used for sows by Gauthier et al. (87) and Gaillard et al. (88).

**Figure 2:**
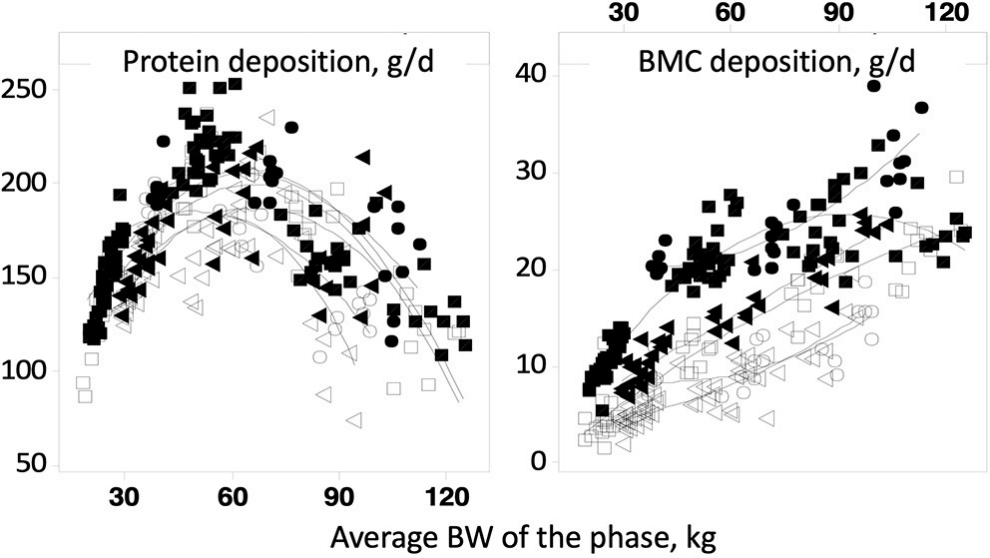
Evolution of protein and bone mineral content deposit as a function of average weight (89).

In all these models, ash deposition strongly correlates with soft tissue gain. However, recent feed trials have shown that this is not the case in growing pigs (Figure 2; 89). Protein deposition increases linearly up to a body weight of about 60 kg, then decreases while bone mineral content deposition increases until the pigs reach slaughter weight (120 kg). These two variables are, therefore, measurements of different physiological processes. In the Jondreville and Dourmad (84) and NRC (20) models, the Ca requirement and the digestible P requirement form a fixed ratio throughout the life of the pig. However, as seen previously, bone growth and soft tissue growth are dissociated, which logically results in a Ca:P requirement ratio that is not the same throughout the life of the animal.

## 4. Strategies to Reduce Phosphorus Excretion

### 4.1. Improved Mechanistic Models to Assess Phosphorus and Calcium Requirements

**Figure 3:**
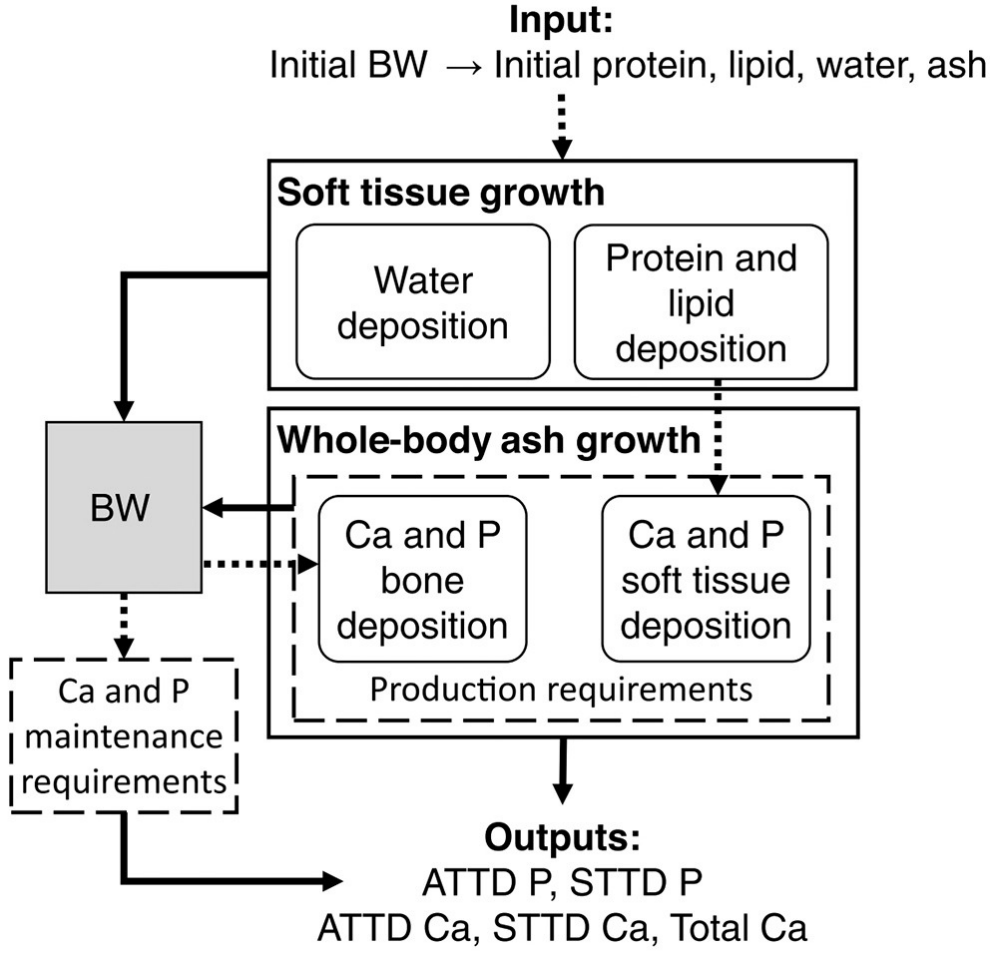
General layout of the proposed mechanistic model predicting total calcium (Ca) and apparent and standardized digestible phosphorus (ATTD and STTD P) requirements of growing pigs (12).

The factorial approach can be integrated in a mechanistic model. The mechanistic models aim to represent the mechanisms of a system. In fact, they connect the underlying mechanisms that control operation of a system. It is, therefore, a matter to meet the conventional notion of requirements (homeorhesis, long-term response) with the response of the animals to inputs [homeostasis, short time scales, (90)]. In a more recent model (11), because of a lack of data, the potential Ca and P depositions were driven by potential protein deposition. But as seen previously, the protein and ash bone depositions are not correlated (89). Consequently, the assessment of Ca and P requirements must consider the fact that changes in skeletal tissue are not directly proportional to lean growth. This is clear when looking at the capacity of P- and Ca-depleted pigs to rapidly replace bone mass through compensatory bone mineralization (see section 4.3). This model has been revised (12) to rectify the no dependency of bone mineral deposition on protein gain by establishing a potential for Ca deposition independent of soft tissue gain, thus allowing P and Ca requirements for soft tissue growth and bone growth to be predicted independently (Figure 3).

This new model estimates apparent digestible Ca and P requirements, which can be converted to STTD or total requirements. The only input required is the initial body weight, from which body protein, lipid, water, and ash (soft tissue and bone) are estimated. Soft tissue growth is currently estimated by applying the principles of van Milgen et al. (91), although other models such as NRC (20) or even user-specific equations tailored to animal growth in a specific setting may be adequate. Estimated protein and lipid gains can be used to assess P and Ca retention in soft tissues (92). In parallel, the Ca requirement for bone is estimated with the bone Ca potential deposition curve presented by the same authors (12). The deposition of P in bone is estimated at a fixed ratio of 2.16 to Ca deposition. The maintenance requirements, equivalent to the obligatory urinary losses, are set at 0.5 mg for P and 2 mg for Ca, per kg of body weight. The sum of the maintenance and growth requirements (of soft tissue and bone) thus provides apparent digestible P and Ca requirements. In fact, the ratio increased with body weight because protein deposition that represents about 30–40% of the body P decreases while bones continue to grow after 70 kg of BW. These can be converted to standardized or total requirements.

Results confirm the need for a non-fixed Ca:P requirement ratio (Table 1). This model has the additional advantage of being adaptable to different production objectives such as 100% or 85% mineralization, without decreasing the share of Ca or P destined for soft tissues. Although a single deposition potential has been established, it will become necessary to consider animal genetics (93, 94) and/or sex in further validations of the model. The sensitivity analysis of the model showed that protein deposition influenced ATTD-P variance by 15% for pigs at 30 kg, 6% at 60 kg, and 1% at 120 kg based on protein deposition variation in previous trials (12). The decrease in the influence of protein deposition on P with BW increase coincides with the linear increase in bone deposition. Moreover, the ATTD-P variance associated with protein deposition at 30 kg shows that animal growth will have a major impact on P recommendations.

### 4.2. Toward More Efficient Degradation of Phytate Phosphorus

#### 4.2.1. Description of Phytases

Phytases, or myo-inositol hexaphosphate phosphohydrolases, are enzymes that hydrolyze phytic acids and release the phosphate groups (55). In growing pigs, there are 4 sources of phytase: (1) the mucosa of the small intestine, (2) microorganisms in the large intestine, (3) ingested plant matter, and (4) exogenous phytase added to the feed. A unit of phytase activity is defined as the release of 1 μmol of inorganic P per minute in a solution containing 5.1 mmol of sodium phytate per liter at pH 5.5 and 37°C (95). Low endogenous phytase activity is observed in the proximal part of the pig intestine, but about 20% of the phytic P would nevertheless be potentially absorbable (72, 76, 77). Some plant raw materials have their own phytasic activity. This one is more or less important according to the ingredient and the part used (14, 84). Phytasic activity is higher in some cereals such as rye, triticale, wheat, or barley than in cereals richer in proteins (19). Plant phytase is sensitive to heat (more than microbial phytases), since its activity is partially or completely inactivated after high temperature treatment (>70°C) such as those for pelleting (18, 84, 96). It is why in INRA-AFZ feed tables (19) two values are given for P digestibility, one which takes account of the effect of endogenous phytase to be used when feed is given as meal, and a second without considering the effect of endogenous phytase to be used when the feed is pelleted. Therefore, the most promising phytase sources remains the exogenous phytase.

#### 4.2.2. New Generations of Exogenous Phytases

The first exogenous phytase was marketed in 1991 in the Netherlands, the first country to introduce strict regulations intended to limit P discharges from pig and poultry farming. The use of phytases then accelerated following the introduction of similar legislation in other countries and the ban on the use of animal byproducts in Europe (18). These enzymes were isolated first from fungi (Table 2), then new techniques allowed the production of phytases by bacteria and yeast, leading to the second generation of phytases. The common commercial phytases are obtained from cultures of *Aspergillus niger, Peniophora lycii* (fungi, 3-phytase), and *Escherichia coli* (bacteria, 6-phytase). In pigs, bacterial phytase has been found to be more effective than fungal phytase (78, 99). This explains why fungial phytases were supplanted in the early 2000s by 6-phytases produced by *Escherichia coli*. Other second-generation phytases obtained from cultures of *Citrobacter braakii, Buttiauxella spp*. and even hybrid forms soon followed (Table 2). Third generation phytases with up to 8 amino acid substitutions in the *E. coli* enzyme have better thermostability (100). The presence of plant phytase reduces the response to added exogenous phytase (18). New generation phytases developed through genetic engineering release more P (101). Exogenous phytases also increase Ca availability (32) but the underlying mechanism remains to be determined. P and Ca digestion in pigs has been modeled, integrating interactions, the different chemical forms, and the effect of phytase (71). Dissociation of Ca phytates at gastric pH is presumed in this model has showed *in vitro* (102). By increasing the proportion of phytate degraded by phytase in the upper digestive tract, less Ca should form insoluble complexes with phytate in the small intestine where pH is favorable and therefore more should remain available for absorption. However, we have not seen validation of this hypothesis *in vivo* and the exact mode of action of phytase on Ca remains unclear, but undoubtedly have an impact *in vivo*. Phytases preferentially release the position 5 and 6 phosphates, which have the highest affinity for cations such as Ca, rather than dephosphorylating phytate completely (103). As a result, the phytase doses that are now commonly used would increase Ca availability more than P availability, at a ratio of about 2, whereas high doses would sustain P release while Ca release reached an asymptote (103).

**Table 2:** Characteristics of some commercial microbial phytases.

**Product**	**Origin**	**Expression**	**Type^a^**	**pH optima**	**IP_**6**_ degradation^b^**	**Year^c^**
Natuphos^®^	A. Niger	A. Niger	3	2; 5–5.5	503	1990
Allzyme^®^ SSF	A. Niger	A. Niger,	3	6		
Finase^®^ P/L	A. Niger	Trichoderma reesei	3	2.5		
Ronozyme^®^ P	Peniophora lycii	Aspergillus oryzae	6	4–4.5	480	2002
Phyzyme^®^ XP	Escherichia coli	Schizosaccharomyces pombe (ATCC 5233)	6	4.5	140	2003
OptiPhos^®^	Escherichia coli	Pichia pastoris	6	3.4; 5.0		2006
Quantum™	Escherichia coli	Pichia pastoris	6	4.5	148	2007
Ronozyme^®^ Hiphos	Citrobacter braakii	Aspergillus oryzae	6	4–5	269	2010
Quantum^®^ Blue	Escherichia coli	Trichoderma reesei	6	4–5	211	2012
Axtra^®^ PHY	Buttiauxella sp.	Trichoderma reesei	6	3.5–4.5	129	2013
Natuphos^®^ E	Hybrid phytase (Hafnia sp., Yersinia sp. et Buttiauxella sp)	A. Niger	6	4–5		2016

*Table 2 is not an exhaustive list and represents only a few of the currently available commercial phytases, adapted from Dersjant-Li et al. (95, 97), and Lei et al. (98)*.

a *3 or 6 phytase*.

b *Phytase activity needed to achieve 50% reduction in IP6, with high buffer volume*.

c *Year of the commercial launch*.

#### 4.2.3. Factors Influencing the Efficiency of Exogenous Phytases

**Figure 4:**
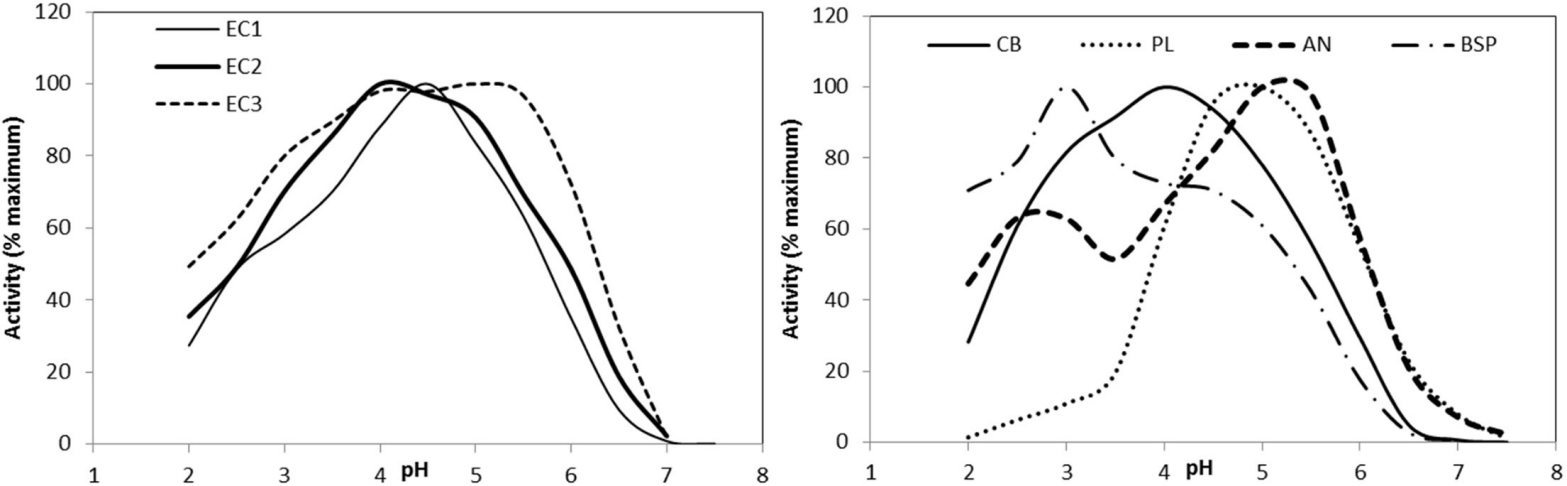
Effect of pH on phytase activity of the phytase products used in the *in vitro* degradation model with EC1: Quantum (AB Vista), EC2: Quantum Blue (AB Vista), EC3: Phyzyme XP (Danisco), CB: Ronozyme Hiphos (DSM), PL: Ronozyme NP (DSM), AN: Natuphos (BASF), BSP: AxtraPHY (Danisco). Reprinted with permission from (105). Copyright 2015 American Chemical Society.

For optimal action, phytic acid must be hydrolyzed upstream from the sites of absorption of P and other minerals such as Ca, Zn, and Fe. P is absorbed mostly in the upper small intestine (5, 51). Hydrolysis in the stomach is therefore ideal, meaning that the enzyme must be sufficiently active at gastric pH (3.5 in young pigs and lower in older animals (104)). Phytase from *A. niger* works well at pH 2 or 5–5.5, but poorly at porcine gastric pH. The optimal pH range of new generation phytases has been lowered and in some cases broadened [Figure 4; (105–107)].

**Figure 5:**
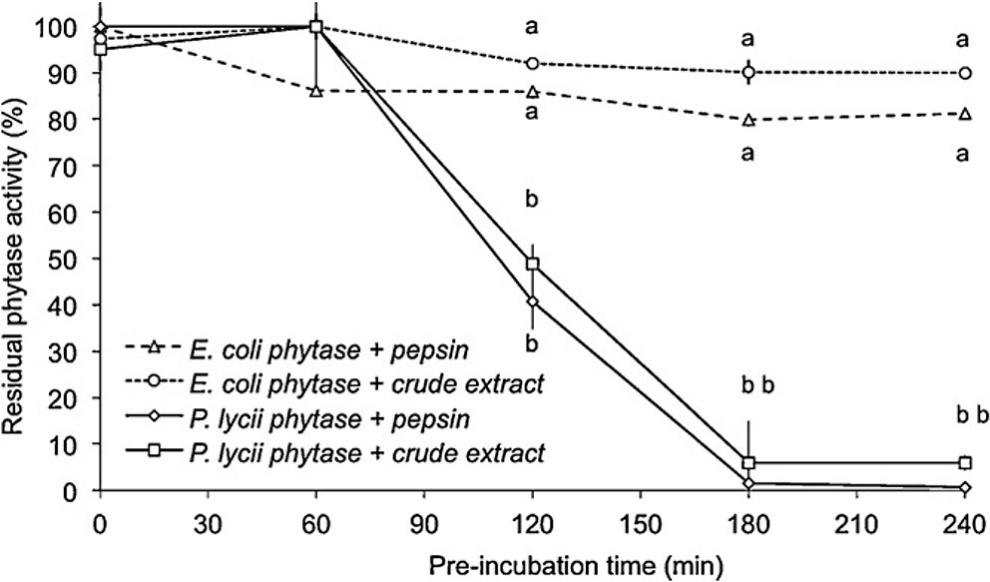
Residual phytase activity of *E. coli* and *P. lycii* phytase after pepsin or gastric crude extract from trout stomach hydrolysis throughout incubation time (0, 60, 120, 180, and 240 min). The incubation was performed by adding 1 FTU phytase to a protease solution with 5000 U from porcine pepsin or gastric crude extract from fish, performed at pH 2.0 (HCl), 16 °C. The results are plotted as the mean ± SE (triplicates). Different letters, for each time, indicate significant differences (P<0.05) between phytases (106).

To limit the loss of activity, phytases must be made resistant to digestive proteases. Second-generation phytases were better in this sense (*P. Lycii vs. E. coli*, Figure 5, 106). After 2 h in contact with pepsin, *E. coli* phytases retained 77% of their initial activity compared to 31% for an *A. niger* phytase (95).

The ideal temperature of activation of the phytase is between 50 and 60°C. On the other hand, high temperature treatments (> 70°C) decrease the phytase activity of the feed (108–110). The second-generation *E. coli* phytases lost thermostability compared to the fungal phytases (98), except for a third-generation phytase from *E. coli* (Phy9X), which is resistant to higher temperatures, up to 75°C (108, 111). On the other hand, increasing the resistance temperature of phytases can lead to a higher optimal temperature and thus potentially decrease their efficacy at normal pig body temperature (around 39°C) (112).

To be the most useful, phytases must preferentially degrade IP_6_ and IP_5_ phytates as quickly as possible. They must, therefore, have a high affinity for the preferred substrate. Second-generation phytases were improved in this sense (112), at least in terms of initial reaction velocity (V_*max*_) *in vitro* with IP_6_ and IP_5_ substrates (105).

Despite the improvement in phytases, it is important to understand that they act on soluble phytates. Therefore, the factors that influenced phytate solubility must be control. Certain minerals interfere with phytates. Cations have an inhibitory power related to their affinities for phytic acid but also the insolubility of the complexes they formed. This is measurable as the amount of mineral that causes phytates hydrolysis to drop by 50% at a given pH (102). The smaller the amount, the more inhibitory. On this basis, the following ranking has been established (102): at pH 6: Fe^2+^ > Zn^2+^= Fe^3+^ >Mn^2+^ >> Ca^2+^>Mg^2+^ At pH 5: Fe^3+^ > Fe^2+^> Zn^2+^ >> Mn^2+^ > Ca^2+^ >> Mg^2+^, representatives of the gut pH in pigs. This inhibitory power represents the affinities of the minerals for phytic acid but also the insolubility of the complexes formed. Reducing the pH to 4, which corresponds to gastric pH, strongly reduces the power of all minerals tested. Iron has the greatest potential for inhibition, but to our knowledge, no study of its effect on phytase effectiveness in animal feed has been published. Regarding Zn and Cu, their supply can be high in piglets with so-called pharmacological levels (2,500 ppm) when used as a growth factor to reduce diarrhea. Zn has a high complexing power, a single Zn cation being capable of binding to two phytic acid molecules (113). The effect of Zn on phytase efficiency has been studied in weaned pigs (6–20 kg). With 1,000 and 3,000 FTU in the diet, zinc oxide at 3,000 mg/kg decreased the Ca ATTD by 6 and 9%, respectively, and the P ATTD by 10 and 16% in pigs weighing 15–20 kg (114). In young pigs weighing 7–13 kg, P release by phytase was reduced by 30% when the dietary Zn content was 1,500 mg/kg (115). The effect of Cu on phytase is less clear. Cu phytates appear to be soluble at neutral pH (113), suggesting no effect. An *in vitro* study of Cu at 62.5 mg/kg and pH 5.5 suggests that P release from phytase may decrease by 2–30% depending on the source of the Cu (116). At 500 mg/kg, the decreases ranged from 5 to 75%. At pH 6.5, the decreases were even more marked but were almost non-existent at pH 2.5. The most likely explanation for these observations would be formation of insoluble phytic acid–Cu complexes at higher pH, which is of some concern given the pH of the porcine gut (116–118). In pigs weighing 6–22 kg, P digestibility was greater with methionine-chelated Cu than with Cu sulfate (118). Chelated Cu would be more stable in the upper gastrointestinal tract and less available to form complexes with phytic acid; thus there would be a better release of absorbable phosphate (116, 119). Tests of the effect of Zn and Cu form and concentration on Ca and P digestibility in pigs weighing 6–22 kg showed that the form of Cu had no effect, while the form of Zn did (117).

**Figure 6:**
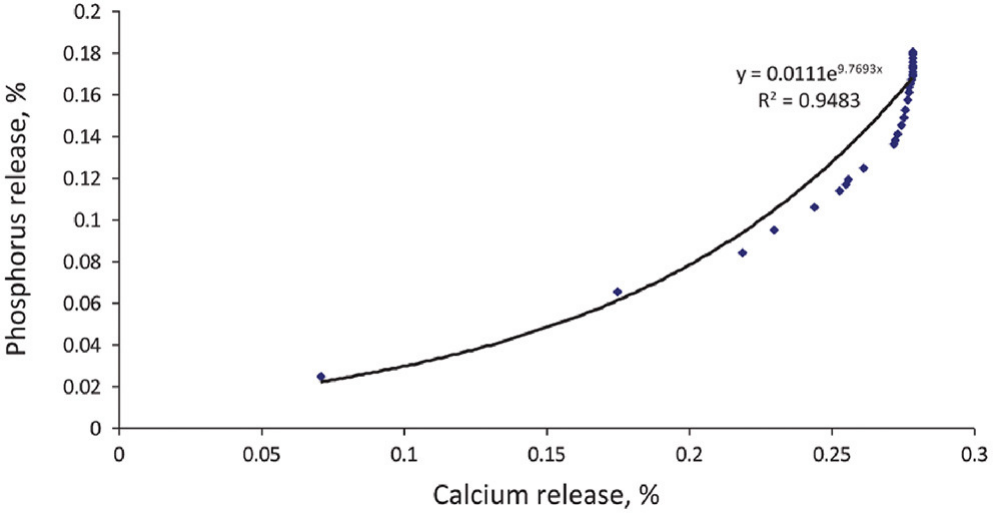
Theoretical relationship between P release from phytate and associated Ca value showing disproportionate extra phosphoric effect with initial destruction of the higher esters (103).

Ca is ranked as less inhibitory but is incorporated into feed at much higher concentrations than Zn, Cu, or Fe. As a result, Ca forms a significant proportion of insoluble phytates, frequently with Zn (120). Because the recent phytases have a higher affinity for IP_6_ and IP_5_, which have higher affinities for Ca, the ratio of Ca:P released using second and third generation phytases is around 2 at 500 FTU/kg (Figure 6) and decreases as phytase activity increases (103, 121, 122). The phytase levels practiced in the field may therefore lead to an increase in the digestible Ca:P ratio. Trials have shown phytase effectiveness to decrease as the Ca:P ratio increases in the feed (123–125) albeit without comparison to phytase-free diets, making it impossible to know whether the effect of Ca was on phytase or on P absorption (126). When the ratio of Ca to total P was increased from 1.2 to 1.8, pigs grew more slowly regardless of the presence of phytase, suggesting a specific effect due to Ca rather than an influence on phytase efficacy in releasing P (78). In trials conducted with P at requirement levels, P digestibility decreased slightly as the Ca:P ratio increased from 1.2 to 1.9 but was indifferent to phytase (79). Furthermore, urinary excretion of P was 5-fold higher at a Ca:P ratio of 1.2, due to the lack of Ca for deposition of P in bone. Nor was any effect of Ca on phytase efficacy found when animals were fed above the P requirement (127). A high Ca:P ratio therefore does not seem to have a direct effect on phytase efficacy in releasing P but rather on P absorption and retention, possibly going so far as to cause a P deficiency and ultimately poorer growth regardless of the presence of phytase (18, 79).

### 4.3. Depletion–Repletion Strategy

**Figure 7:**
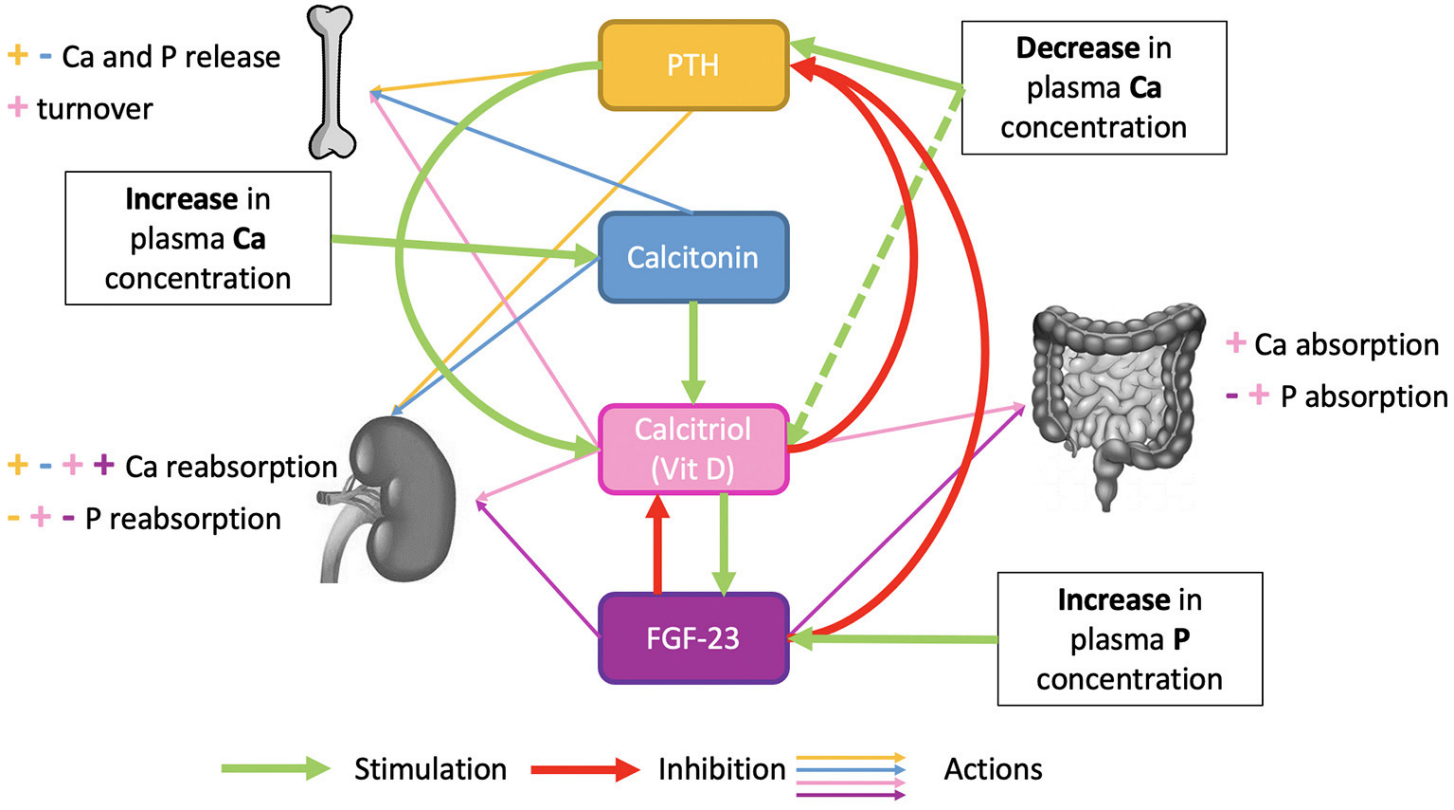
Hormonal regulation of phosphocalcic metabolism.

Animals have a survival strategy to overcome some mineral deficiencies by enhancing digestion and increasing the efficiency of utilization of the deficient nutrient (128). In several species, dietary restriction of Ca and P results in increased intestinal absorption, renal reabsorption, and deposition and mobilization in bone tissue (1). The effects of dietary Ca restriction and recovery processes on bone metabolism were studied decades ago in rats (129–131) and humans (132). The findings suggest that bone has ways of replenishing losses due to the use of mineral reserves and that parathyroid hormone and vitamin D play a role in the mechanisms. Bone accretion, intestinal absorption, and renal reabsorption of minerals are under hormonal regulations described in Figure 7.

The Ca depletion–repletion strategy is already used to prime dairy cows for high Ca demand during early lactation (133) and to prevent milk fever. By feeding a Ca-deficient ration for a few weeks before the start of lactation, regulatory mechanisms that maintain blood Ca levels (increased intestinal absorption and renal reabsorption) are activated (133, 134). A few days before calving, when the demand for Ca becomes very high, the cows then receive more Ca (134), and the shortfall between the requirement and Ca absorbed is smaller because of the effect of priming on parathyroid hormone. The animal is also better prepared to draw upon bone Ca as needed to maintain blood Ca levels and thus prevent milk fever. Similar regulatory mechanisms allow maintenance of P levels, and these can be exploited to increase dietary P utilization in growing animals and hence the sustainability of livestock farms from the environmental perspective. The idea underlying the depletion–repletion strategy is therefore to trigger regulatory mechanisms during the depletion phase to induce an increase in P utilization efficiency without affecting growth performance (1). In the case of P and Ca, the mineral content of the body or of a specific bone is monitored using X-ray absorptiometry [DXA, (135)]. During depletion in growing pigs, body bone mineral content continues to increase, but bone accretion is decreased compared to control pigs, leading to reduced bone mineral content.

When P supply is intentionally below the estimated requirement of the animals, the level of Ca is generally decreased at the same time to avoid the deleterious effects of a high digestible Ca:P ratio on P absorption. When animals thus primed are fed the repletion diet, which provides P at least at the requirement level, the deficit overcome. This allows an overall reduction in dietary P intake during the rearing phase. Depletion–repletion studies of growing animals such as pigs (8, 10, 136) and chickens (137–141) led to effectively increasing P utilization and limiting excretion without compromising animal well-being and performance. Some authors (9, 142) have focused on improvements to bone health; these studies have led to better understanding of the deleterious effects of short-term dietary Ca deficits during growth on long-term bone mineralization. The main trials performed with pigs are summarized in Table 3.

**Table 3:** Effect of depletion–repletion strategy on bone mineralization of growing pigs.

			**Depletion period**										**Repletion period**							
**Article*^a^***	**Measurement**	**Phase**	**Sequence*^b^***	**BW, kg**	***p*-value*^c^***	**Days*^d^***	**P*^e^*, %**	**Ca*^e^*, %**	**Bone*^f^*, %**	***p*-value*^c^***	**Bone accretion*^g^*, %**	***p*-value*^c^***	**Sequence*^b^***	**Days*^d^***	**BW, kg**	***p*-value*^c^***	**Bone*^f^*, %**	***p*-value*^c^***	**Bone accretion*^g^*, %**	***p*-value*^c^***
1	BMC total body (DXA)	1	L	34	ns	28	-31	-39	-34	<0.001	-62	<0.001								
		2	CL	67		28	-42	-22	-25		-48		LC	28	64	0.03	-17	<0.001	+2	ns
			LL	66	ns	56	-42	-22	-25	<0.001	-45	<0.001								
		3	CCL	102	ns	28	-34	-13	-14	0.002	-31	<0.001	CLC	28	100	ns	-13	0.006	+8	ns
			LLL	100		84	-34	-13	-33		-23									
		4											CLCC	56	129		-3		+11	
													CCLC	28	134	ns	+1	ns	+29	<0.001
2	BMC of the L2 to L4 vertebrae	1	Low	46	ns	28	-40	-30	-29	<0.001										
		2	Con-Low	72		28	-40	-46	-24		-53		Low-Con	28	70	<0.01	-9	0.007	+17	0.005
			Low-Low	77	ns	56	-40	-46	-30	<0.001	-36	<0.001								
		3	Con-Con-Low	104		28	-40	-33	-2		-18									
			Con-Low-Low	101		56	-40	-33	-16		+1		Low-Con-Con	56	99		-1		+15	
			Low-Low-Low	106	ns	84	-40	-33	-18	<0.001	0	ns	Low-Low-Con	28	103	ns	-7	ns	+56	<0.001
3	Ash of the 3^rd^ and 4^th^ metacarpus	1	L	48	<0.001	59	-47	-29	-9	<0.05										
		2	LL	91	<0.05	131	-45	-30	-7	<0.05			LH	72	99	ns	-1	ns		
			HL	100	ns	72	-45	-30	-1	ns										
4	BMC total body (DXA)	1	L			28		-54	-66	<0.01										
		2	LL			71		-54	-62	<0.01	-60	<0.01	LH	43					+3	
			HL			43		-54			-59	<0.01								
5	Femur ash	1	DD-	12	ns	10	-60	-53	-19	<0.01										
		2	DD+ HCaPhyt-	21		25	-32	0	-10				DD- HCaPhyt+	25	21		1			
			DD+ LCaPhyt-	21		25	-32	-37	-7											
			DD- LCaPhyt+	21		35	0	-34	-1											
			DD- HCaPhyt-	21		35	-32	0	-17											
			DD- LCaPhyt-	21		35	-32	-37	-19											
6	BMC total body (DXA)	1	Phyt	71	<0.05	39	-40	-40	-17	<0.001	-23	<0.01								
		2											Phyt-Phyt	27	108	ns	+3	ns	+47	<0.05
		3											Phyt-Phyt-Phyt	55	130	ns	+7	ns	+4	ns

a *Article 1 : Gonzalo et al. (136), article 2 : Létourneau-Montminy et al. (8), article 3 : Varley et al. (10), article 4 : Aiyangar et al. (9), article 5 : Létourneau-Montminy et al. (79), article 6 : Lautrou et al. (143)*.

b *Sequences of depletion and repletion as named in the original articles*.

c *p-value of the statistical analysis of the control vs. the studied group, for the variable of the previous column*.

d *Duration of the depletion or repletion*.

e *P or Ca depletion against the control*.

f *Difference of the state of the bone at the end of the phase between the control vs. the studied group, according to the measurement*.

g *Difference of the bone accretion measurement between the control and the studied group*.

When Ca is deficient, the Ca regulation calls for parathyroid hormone, which is a hypercalcemic hormone that increases dietary Ca utilization, but with a concomitant hypophosphoremic effect due to renal excretion of P (144). Ca deficiency must therefore be avoided. In growing pigs, it has been found to reduce the expression of genes related to P reabsorption in the kidney, favoring P excretion in urine (145). P depletion in the range of 30–40% and slightly lower for Ca induces demineralization of the same order in the whole body and vertebrae as measured by DXA (Table 3). The trial conducted by Aiyangar et al. (9) shows greater demineralization with a higher Ca deficiency. The metacarpus appears to demineralize less (5–10%) than the whole body or vertebrae (10), whereas the femur responds like the whole body. Bone reserve depletion measured thus depends primarily on the degree of dietary depletion and on the bone region studied.

**Figure 8:**
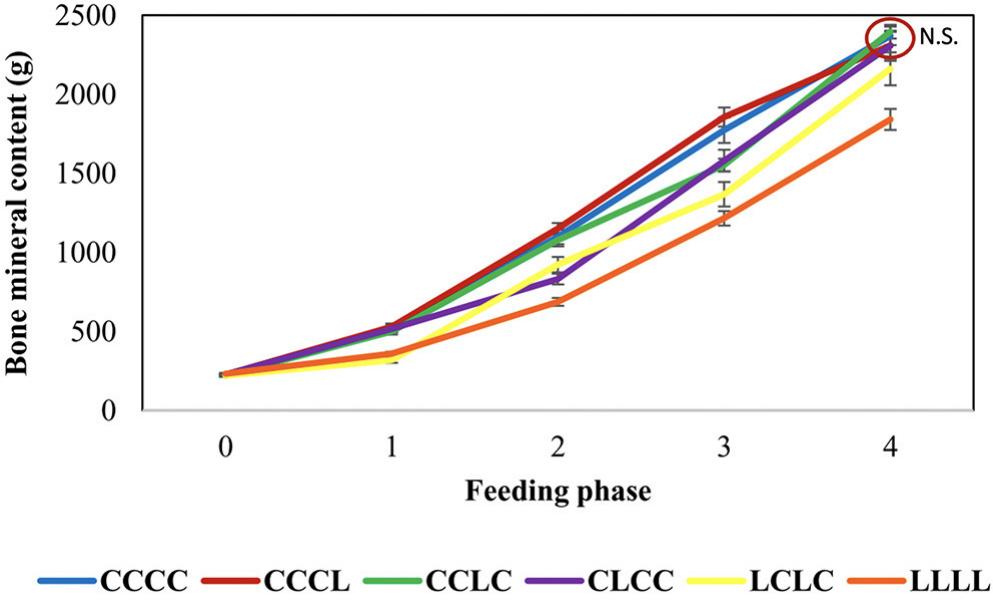
Body bone mineral content of growing pigs feeding depletion–repletion diets (145).

Several studies have shown that this strategy works and increases bone mineral content (BMC) gains and digestible P utilization when animals are fed a repletion diet (at requirement or above). The gain of BMC in L2 to L4 vertebrae in depleted animals exceeded those in non-depleted control pigs by 56% during a first 28-day repletion phase and 15% during a second repletion of the same length (8) and by 29% after a 28-day repletion and 11% after a 56-day repletion in another study (Figure 8; 136). The corresponding increases in digestible P utilization estimated as deposition vs. intake were 20–50% with bone deficit recovery in 28–56 days for the whole body and in 28 days for vertebrae. The shorter time for vertebrae could be due to their high percentage of trabecular tissue, which is more sensitive than cortical tissue to mineral deficiencies (146). Furthermore, in pigs, bone mineralization is faster in the trunk from 3 to 30 kg of BW than in other parts of the skeleton (147). In a study using the common dosage of 750 FTU/kg without phosphate, thus 40% below the requirement, the deficit was recovered in 27 days on the repletion diet with a 47% increase in whole body BMC gain (143). Overall, the depletion–repletion strategy reduced dietary phosphate use and P release by about 40%. In contrast, the repletion diet has failed to restore bone mineralization in at least two porcine studies (9, 142).

Phosphocalcic regulations occur in the gut, kidney, and bone (32, 148). Ca absorption may increase by 27% upon repletion compared to control animals receiving the same feed (8). Osteocalcin, derived from newly synthesized bone and thus an indicator of osteoblastic activity and hence increased bone accretion (149), has been found to increase during the repletion phase (10). The physiological mechanisms underlying animal responses to the depletion–repletion strategy remain poorly understood. It, nevertheless, appears that adequate bone mineralization and growth performance can be achieved at decreased P intake and excretion through improved P utilization.

## 5. Mitigation Strategies: Comparison and Perspectives

The mitigation of the environmental footprint of P in pig production both refers to the optimization of the use of phosphates, which are a non-renewable resource that must be extracted and transported, and to the minimization of its excretion especially in regions with high production density. Some strategies of mitigation have been proposed in the previous sections.

The potential for decreasing P excretion with phytase is well known (18). Its potential depends mainly on a precise nutritional matrix. First, a precise estimation and utilization of the P matrix is crucial. The Ca matrix has recently been shown to be of great importance because, on the one hand, an excess of soluble Ca can decrease the P digestibility (78) and, on the other hand, in case of Ca deficiency, the P is not retained and is excreted in the urine (79). In recent trial with microbial phytases (500 FTU), Lagos et al. (150) showed a drop of 37% in the total P excretion, and a reduction of 6.1 g/kg monocalcium phosphate supply, for 60 kg BW pigs. Besides, Almeida and Stein (55) showed that the total P excretion decreases up to 51% with 685 FTU at 15 kg of BW, and monocalcium phosphate supply is reduced up to 8 g/kg.

The depletion–repletion strategy also led to a decrease of the phosphate input and the P excretion. In Gonzalo et al. (136)'s trial, a depletion period of 28 days (L) resulted in a decrease of P supply from 8 to 12%, and 2 separate depletion periods of 28 days (LCLC, C being a 28 days phase of feeding control diet) resulted in a reduction of 12% of P input. The excretion of P in the CLCC, CCLC, and LCLC groups decreased of 15, 13, and 16%, respectively, compared to the control. The decrease of P excretion was greater than the decrease of P input thanks to animals utilizing P more efficiently during the depletion and the repletion periods. With P total collection of feces and urine, Létourneau-Montminy et al. (8) showed that a depletion of 56 days can lead to a P excretion decrease of 19% with a diminution of P intake of 23%. As seen previously, few authors tested depletion–repletion strategies on growing pigs and the results differ in terms of bone mineralization compensation (145). There is a lack of data to precisely defined an ideal strategy of depletion repletion (depletion duration, age, intensity). The study of underlying mechanisms, such as hormonal regulations, will certainly help to reach this objective to reduce phosphate input without compromising bone mineralization and to apply this strategy on farm. Nevertheless, a reduction of 15–20% of both phosphate use and P excretion may be achieved with depletion–repletion strategy.

The strategy of depletion–repletion can also be combined with phytase. Lautrou et al. (143) tried to evaluate the effect of a zero phosphate diet on the growth of pigs and the environment. The use of phytase did not meet the full extent of the P requirements for pigs during the first growing phase of 39 days, but phytase provided enough P during the 2 next phases. This strategy led to a drop of 66% in P excretion during the 2 first phases (the data are not available for the last one), and a reduction in monocalcium phosphate supply of 18.71, 9.52, and 7.17 g/kg in Phases 1, 2, and 3, respectively. This trial showed that there is an opportunity to feed growing pigs from 30 to 130 kg without adding any mineral phosphates. This success has to be confirmed and always requires a well-defined phytase matrix, particularly to mitigate the risks associated with the depletion phase.

In a simulation, Pomar et al. (151) showed that precision feeding, a strategy in development that allow to feed pigs with diets tailored daily to each individual's nutrient requirements, could reduce P excretion by 38%. A recent study compared the P excretion of pigs under conventional or precision feeding (152). The individual and daily feeding system (based on estimated lysine requirement) led to a decrease of 27% in P excretion compared to the group phase feeding system. In this trial, phytase was used but not compared with a control without phytase. The combination of precision feeding with phytase and a depletion–repletion strategy has not been tested yet, but after the synergy observed with the phytase and depletion–repletion strategy, combining these 3 methods seems a promising strategy that could lead to an even greater reduction of P excretion.

## 6. Conclusions

This review has shown that it is still possible to improve P utilization in swine and to improve the sustainability of the industry by mitigating phosphorus' impact on the environment. The first step is to precisely estimate the P and Ca content of feedstuffs and each animal's total diet. The second step is to use a robust multicriteria modeling approach to establish animal requirements. The new generation of phytases may provide a strategy to increase P utilization by pigs by providing a precise estimation of the equivalences and interfering factors and maximizing the solubility of phytates. A depletion–repletion strategy to prime animals to make them more efficient is also promising, but still requires testing to refine it and better understand the underlying mechanisms. Finally, precision feeding, a strategy in development that permits feeding pigs with diets that are tailored daily to each individual's nutrient requirements, shows possibilities to reduce more P excretion, and will undoubtedly be employed once the P requirements will be well defined by a robust modeling approach.

## Author Contributions

ML wrote the original draft. AN, J-YD, CP, PS, and M-PL reviewed the article and add new ideas and participate to improve the structure of the document. All authors contributed to the article and approved the submitted version.

## Conflict of Interest

The authors declare that the research was conducted in the absence of any commercial or financial relationships that could be construed as a potential conflict of interest.

## Publisher's Note

All claims expressed in this article are solely those of the authors and do not necessarily represent those of their affiliated organizations, or those of the publisher, the editors and the reviewers. Any product that may be evaluated in this article, or claim that may be made by its manufacturer, is not guaranteed or endorsed by the publisher.
